# Sick leave and the impact of job-to-job mobility on the likelihood of remaining on the labour market - a longitudinal Swedish register study

**DOI:** 10.1186/1471-2458-14-305

**Published:** 2014-04-03

**Authors:** Karin Nordström, Kerstin Ekberg, Tomas Hemmingsson, Gun Johansson

**Affiliations:** 1 National Centre for Work and Rehabilitation, Department of Medical and Health Sciences, Linköping University, Linköping 581 83, Sweden; 2 Unit of Occupational medicine, Institute of Environmental Medicine, Karolinska Institutet, Stockholm, Sweden; 3 Centre for Social Research on Alcohol and Drugs, Stockholm University, Stockholm, Sweden

**Keywords:** Job-to-job mobility, Sick leave, Vocational situation

## Abstract

**Background:**

Change of job could be a strategy in vocational rehabilitation when return to the original job is not possible, but research is very limited concerning the effects of job mobility on the future vocational situation. The aim of the study was to investigate whether job-to-job mobility affects the likelihood of remaining on the labour market over time among persons who are employed and have experienced long-term sick leave.

**Methods:**

In a longitudinal register study, cohorts from three base years (1994, 1999 and 2004) were created, based on the Swedish population who were 20–60 years old, had sickness allowance insurance, and were employed in the base year and the following year (n > 3,000,000). The likelihood that individuals on long-term sick leave were employed later depending on whether or not they changed workplace during the present or next year of long-term sick leave was analyzed using logistic regression analysis. Age, sector, industry, children, marital status, education, income, rate of sick leave and earlier sick leave and earlier mobility were taken into consideration.

**Results:**

Women with more than 180 days’ sick leave who changed workplaces were more likely to have a job later compared with those who did not change jobs. For men, the association was statistically significant with 1994 and 2004 as base years, but not in the cohort from 1999.

**Conclusions:**

The present study indicates that for those on long-term sick leave that changed workplaces, the opportunities to stay on the labour market might increase. However, the study has methodological limitations and the results for men are ambiguous. We do not therefore have enough evidence for recommending job change as a strategy for vocational rehabilitation.

## Background

In November 2010, 36,535 women and 20,232 men had 180 or more days of sick leave in Sweden, corresponding to approximately 1.7% and 0.9% of the employed population respectively (http://www.forsakringskassan.se/statistik/sjuk/sjuk_rehabiliteringspenning/sjukochrehabsjukpenning/ and http://www.ssd.scb.se/databaser/makro/Produkt.asp?produktid=am0207) and contributing to the majority of disbursed days of sick leave.

The costs of disbursed sickness allowance are high for the community. In 2010, the cost of sickness cash benefits was approximately EUR 2,071 million and the expenditure on sickness and disability amounted to just under 4% of GNP in Sweden [1]. Lost competence that has to be replaced may also be costly for employers. For the individual, long-term sick leave may have a negative impact on health and finances [2-6]. Long-term sick leave increases the likelihood of losing affiliation with the labour market in subsequent years [7-10] and reduces the probability of gaining new employment [11,12]. Poor health, measured as hospital admission, has been found to increase the probability of exit from employment by 48% among men and 43% among women [13].

According to the Swedish Social Insurance Administration, 62% of the individuals who were on long-term sick leave (60 days or more) in 2006 were assessed as having full working capacity 1 year later. Eighty-one percent of these were employed or self-employed 1 year later, and 15% were unemployed. In a study from 2009 of individuals on sick leave with chronic occupational back pain in Denmark, Germany, Israel, the Netherlands, Sweden, and the United States, the degree of return to work after 2 years ranged from 22% in Germany to 62% in the Netherlands. In Sweden, the degree of return to work was 39% 2 years after the first day of sick leave [14].

Accommodation at work, such as changing the workplace, the equipment, the conditions or the environment with the purpose of removing barriers for return to work, seems to be effective in facilitating return to the same workplace after periods of sick leave [15-18]. However, such accommodation is not always feasible. An example could be an individual suffering from rheumatism with physically heavy work where no co-workers are available to allow changes to the work. Furthermore, many do not return to their original employment despite accommodation at work due, for example, to cooperation problems among key stakeholders, lack of motivation to return from being sick listed or negative reactions on return to work from supervisors or the work group [15,19,20].

Change of job is discussed as a central strategy in vocational rehabilitation when return to the original job is not possible [21]. A change could mean improved ability to work through a better match between the individual’s capacity and the demands of the job. A change could also promote health when the individual is dissatisfied with his or her present job. According to the person–environment fit model, strain develops when the individual’s needs or abilities do not match the supplies or demands of the job. Such strain can lead to illness [22]. A change to a job that better matches the individual's abilities and needs could relieve the strain [23]. One type of mismatch between the individual and the job is being locked in; that is, being in an unwanted occupation and/or workplace and experiencing a real or perceived lack of alternatives [24,25]. A locked-in situation is associated with having health problems and being on sick leave [26]. Change of work in these situations is associated with increased job satisfaction and fewer conflicts at work [24].

Mobility on the labour market is a complex concept. One form of such mobility is job-to-job mobility. Job-to-job mobility may be characterized as change of employer/company, change of workplace, and/or change of profession/work tasks. This study concerns change of workplace.

A strong association between health and mobility and between employment and unemployment has been found, but there is a weak association between health and occupational mobility [11,13]. According to a report from the European Union, 8.8% of the general Swedish population changed jobs during 2005. The United Kingdom had the highest proportion that had changed jobs at 22.9% and Greece had the lowest proportion of job changers at 5.6% [27]. A report on job mobility in the Nordic counties in 2010 showed that Sweden has had a comparatively low rate of mobility compared to the other Nordic countries [28].

Mobility decreases with age [28-31]. Mobility also differs between occupations. For example, managers in Sweden change jobs to a greater extent than other occupational groups [28]. Mobility also varies with branch code (see Methods section). In Sweden, individuals with a high level of education move to a greater extent than individuals with a low level of education [28,32]. Married people are less mobile than those who are single or cohabiting. Women with children are in general less prone to change jobs than men. In the Nordic countries, the type of contract and the number of working hours per week have been found to have a strong effect on mobility. Being in temporary employment and/or working part time increases the probability of a job change [28]. These conditions may also affect return to work and are therefore considered in this study. The association between mobility and employment may also be affected by health selection, that is, individuals with better health may have better opportunities to change jobs and better conditions to stay employed.

For employees on sick leave, a job change may thus lead to improved work ability, health and the possibility of remaining employed, but research is very limited concerning the effects of job mobility on the future vocational situation. To our knowledge, there are no previous studies on whether a change of job increases the possibility of remaining on the labour market for individuals who have been on long-term sick leave.

### Aim

The aim of this study was to investigate whether job-to-job mobility affects the likelihood of remaining on the labour market among persons who have experienced long-term sick leave and whether this likelihood differs between men and women. In addition, this study explored how this association between job-to-job mobility and labour market situation differs with regard to job change among employees without or with fewer days of sick leave.

## Methods

### Ethics statement

The study has been approved by the Ethical review board in Linköping, Dnr:169–09.

### Sample and design

In this cohort study, data were collected from LISA, a longitudinal database of Swedish registers of social insurance and labour market studies established by Statistics Sweden, the National Insurance Administration and the Swedish Agency for Innovation Systems (VINNOVA). Permission to use the database was given by Statistics Sweden. The database is updated annually with a 3-year delay and includes all registered residents aged 16 years and older for the period 1990 to 2009.

**Figure 1:**
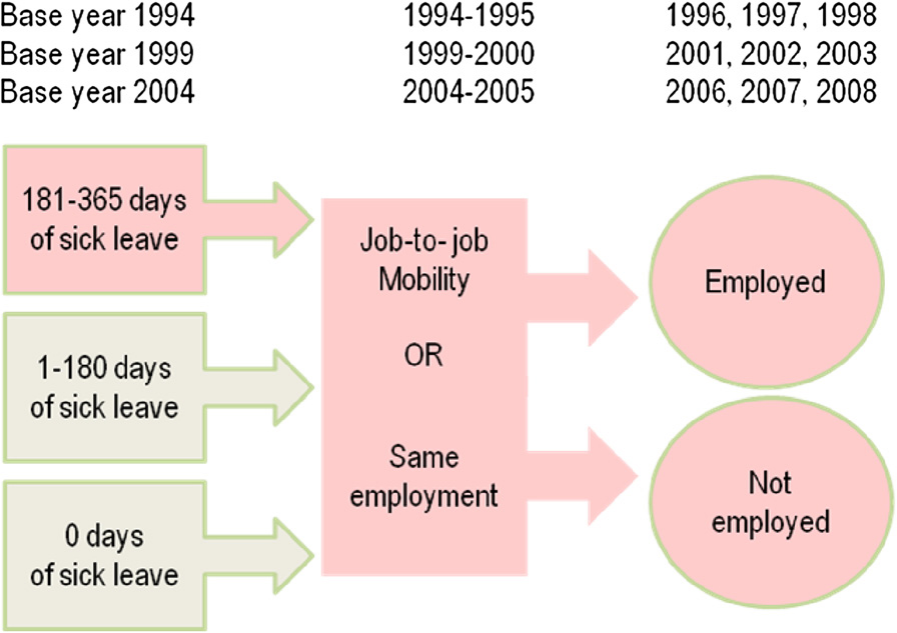
The design of the study.

In a first step, three cohorts were created based on the Swedish population who in 1994, 1999 and 2004 were between 20 and 60 years old, had sickness allowance insurance and were employed at the base year and the following year (see Figure 1). Those who were self-employed at the base years and the next year were excluded from the study as they differ from employed persons in having a significantly lower level of sickness absence and other conditions in the social security system such as a longer qualification period before getting sick pay [33]. Based on these criteria, the total number of females varied between 1,448,972 (1994) and 1,503,397 (2004). Comparable figures for males were 1,482,668 (1994) and 1,568,961 (2004).

The individuals were followed for 4 years; emigrants, early retirees (<65 years), and deceased individuals were excluded.

The individuals in each cohort were divided in regard to sickness absence in the base year. In order to be able to investigate the specific research question of this paper, the long-term sick, defined as those having 181 days of sickness absence, is the group in focus. However, we have also analysed those having between 1 to 180 days of sickness absence and those lacking absence at each base year for comparison.

Only between 18.5% and 49.9% of long-term sick men and women matched our criteria of having employment both at the base year and at the year after (Table 1).

**Table 1:** Number of individuals stratified according to days of sick leave at the base year

**Base year**	**Gender**	**Days of sick leave at base year**	**(A) No. aged 20–60 years at base year**	**(B) % of A. aged 20–60 years employed at base year and the next year (excluded: self-employed, early retired)**	**Lost to follow-up year 1 ** **(% of B)**	**Lost to follow-up year 2 ** **(% of B)**	**Lost to follow–up year 3 ** **(% of B)**
1994	Females	0	1907328	64.0	−0.2	−0.6	−0.9
		1–180	337787	63.2	−0.4	−0.7	−1.1
		181–365	44151	32.3	−1.3	−2.4	−3.3
	Males	0	2092984	64.1	−0.4	−0.8	−1.3
		1–180	240180	55.5	−0.6	−1.0	−1.8
		181–365	38156	23.2	−1.4	−2.6	−3.7
1999	Females	0	1914587	65.5	−1.6	−1.8	−2.1
		1–180	318709	67.9	−0.7	−1.1	−1.4
		181–365	66897	34.7	−1.1	−1.8	−2.5
	Males	0	2135039	68.7	−1.6	−2.0	−2.3
		1–180	199384	62.1	−1.2	−1.7	−2.1
		181–365	43000	18.5	−1.2	−2.0	−3.0
2004	Females	0	1931931	65.7	−0.3	−0.5	−0.8
		1–180	294135	68.1	−0.3	−0.6	−0.9
		181–365	66772	49.9	−0.7	−1.1	−1.7
	Males	0	2168048	66.8	−0.4	−0.8	−1.2
		1–180	170194	61.2	−0.5	−1.1	−1.7
		181–365	38949	41.5	−1.0	−1.9	−2.7

In the cohort from 1994, year 1 = 1996, year 2 = 1997 and year 3 = 1998. In the cohort from 1999, year 1 = 2001, year 2 = 2002 and year 3 = 2003. In the cohort from 2004, year 1 = 2006, year 2 = 2007 and year 3 = 2008.

### Institutional background

In 2004, there was no maximum duration of sickness benefit in Sweden; in practice, it was possible to be on sick leave for several years. Also, there were no set time limits for getting sickness compensation. Sickness benefit is usually close to 80 percent of the calculated annual income and sickness compensation amounts to 64 per cent of the assumed income. An employee on sick leave could be fired under the same circumstances as an employee not on sick leave. In both cases, there must be objectively based reasons such as shortage of work. The employer has a responsibility to reassign the employee within the company if possible [34]. In 2004, there was also a National Insurance Act stating that the employer shall ensure that actions are taken for active rehabilitation [35].

After the first 14 days of sick leave, the employer does not fund sickness benefit, thus it is possible to change jobs during sick leave. Traditionally in Sweden, employers can get temporary grants from the Swedish Public Employment Service to partly cover salary costs and work appliance as an incentive to hire disabled individuals.

### Variables

#### Prediction variable

Job-to-job mobility was defined as a change of workplace identity the year following the base year (Figure 1). Information on workplace identity was based on payment of salaries during a week in November and was collected from administrative registers at the Swedish Tax Agency. Employers in Sweden have a duty to report annually to the tax authorities the salaries paid and the workplace identification numbers for all employees. A workplace is any address, dwelling unit or group of dwelling units where some sort of economic activity is carried out, with at least one employee working at least 20 hours per week. A company can have several workplaces, but a workplace can only belong to one company.

#### Outcome variable

Being employed or not being employed is the outcome variable and is based on being gainfully employed 4 years after the base year (Figure 1). Individuals were classified as gainfully employed if they had worked for at least 1 hour per week in November, and otherwise as non-employed. This is based on information that employers are obliged to provide to the Swedish Tax Agency. During 1998, 190 individuals were found to be classified as gainfully employed despite 12 months of full-time sickness compensation and they were treated as not being in active work and were reclassified as non-employed. In 2003, 945 individuals were classified as gainfully employed despite 12 months of sickness benefits and 1023 individuals were reclassified in 2008. This reclassification only applies to individuals with full time sickness compensation for the whole year, and not to those with part-time sickness compensation. The reclassified individuals most likely have no real attachment to the labour market; the fact that they still have an employment number might be due to retained holiday compensation. Some of those classified as employed also had other sources of income such as economic support, unemployment benefits or sickness benefits to some extent during the year or that they had participated in some form of labour market intervention. In an attempt to control the predictive validity of the outcome variable, the occurrence of alternative sources of incomes was compared between those classified as employed and those classified as non-employed. Individuals classified as non-employed had other income sources to a much greater extent, such as unemployment benefits. This implies that the measurement of employment is valid.

#### Stratification variables

● Data were stratified on sick leave and were collected from the Swedish Social Insurance Administration. In Sweden, the employer pays for the first 14 days of sick leave, apart from the first day, which is a qualifying day of sickness. The Swedish Social Insurance Administration pays from day 15. As information about days of sick leave is collected from the Swedish Social Insurance Administration, this means that one registered day of sick leave corresponds to 15 days of sick leave (14 + 1) in reality. In Sweden, sickness allowance can be given for a whole day or 0.25, 0.50, 0.75 of the working day. One way to describe sick leave based on sickness allowance is to ascribe 1 day of sick leave regardless of whether it is given for a whole or part of a day (gross days). An alternative way, which is used here, is to recalculate allowance for part of a day to full days (net days) so that 1 day of sick leave can mean e.g. 1 day of full sick allowance, 2 days of half-sickness allowance (0,50) or 4 days of 0,25 sickness allowance.

The population was stratified into three categories based on days of sick leave registered by The Swedish Social Insurance Administration in the base year: 0 days; 1–180 days; and 181–365 days. As pointed out above, the category ‘0 days’ encompasses days of sick leave lasting 14 days or less which are paid by the employer. The population was also stratified according to gender.

### Potential confounders

All confounders were collected from the base year (1994, 1999, 2004). Age, Sector and branch of employer, children, marital status, income, education, sick leave before the base year, job change before the base year and rate of sickness absence were considered as possible confounders. All confounders had a statistically significant univariate correlation with employment status 3 years later (p < 0.05). The variance inflation factor (VIF) scores in this study ranged between 1.01 and 1.37, which can be considered acceptable [36].

● Age: Age was calculated from the year of birth based on the Social Security Number and was used as a continuous variable.

● Sector of employer and branch of employer: Information about sector was collected from Statistics Sweden’s Business Register. Statistics Sweden is assigned by the Swedish parliament to maintain a register of enterprises, government offices and organizations and their workplaces. The sectors are divided according to the standard classification by institutional sector. The institutional sectors correspond to ESA sectors (European System of National and Regional Accounts) with one difference: the general government sector has been divided into three sectors. Which ESA sector a unit belongs to depends on the type of activity, its function and what is being produced. Based on the institutional sector code, each company is assigned a sector code. These sector codes are grouped in ten categories. In this study, these categories were merged into municipal (primary municipal administration, county councils and municipally-owned companies and organizations), public (public administration, public utility companies and government-owned companies and organizations), private (stock corporations and other non-public companies) and other (any other organization). Information about sector is connected to the company where the individual is employed. Information about company is collected during a week in November, so if the individual has several jobs during the year, it is the sector of the company from which the individual got the major part of his or her paid salary in November that is registered. Information on branch was collected from Statistics Sweden’s company register with information from the Swedish Tax Agency. Based on the activities carried out, every company is assigned one or several activity codes according to the Swedish Standard Industrial Classification. This classification is based on the EU’s recommended standard NACE (Nomenclature statistique des activités économiques dans la Communauté européenne) [37]. The short, two-digit version was used.

● Children and marital status: Information about number of children was collected from national registrations conducted by the Internal Revenue Service. In the register, children are connected to their family identity and are registered at the same property as the parents. Children with divorced parents are connected to the same family identity as the parent with whom they are nationally registered. The other parent is registered as childless unless he or she is living with another parent with children nationally registered at the same address. Family identities are created by the social security number of the oldest individual of a maximum of two generations who are connected to each other (married, registered partners, cohabitants with common children, parents or foster parents). Marital status is categorized as unmarried, married/registered partner, divorcee or widow/widower.

● Income and education: Income and education were used as indicators of socioeconomic status. Income refers to disposable income and is the individual’s contribution to the household income. Disposable income is what is left of salaries and benefits from the state and local authorities after taxes and deductions. Income was used as a continuous variable. Information on education was collected from the Swedish Register of Education, which records the highest education registered from all formal education units in Sweden for each individual each year. Education is classified according to the Swedish Nomenclature of Education (SUN), which is adjusted to meet the International Standard Classification of Education (ISCED).

● Sick leave in the year before the base year: The study works with days of sick leave during the year before the base year (see section on stratification variables for description).

● Mobility between the previous year and the base year: The study works with mobility between the previous year and the base year (see section on prediction variables for description) in order to check individuals with a pattern of frequent job changes.

● Rate of sickness absence: The rate of sickness absence was calculated by dividing the sum of net days of sick leave at the base year with the sum of gross days. One gross day of absence could mean being absent 100%, 75% or 25% of the day (see description of net and gross day above under Stratification variables). With net days, one day of absence means 100% of a day, two days with 50% absence or four days with 25% absence. The rate between net days and gross days was used to check whether part-time or full-time sick leave affect the association between job change and future labourmarket situation.

### Analysis

Differences in being employed or not 3 years later with respect to job change were investigated using Pearson’s chi-squared test. Logistic regression analysis was used to calculate the likelihood (odds ratio (OR)) that individuals sick listed for more than 180 days were employed 2, 3 and 4 years later depending on whether or not they changed their job between the base year and the next year. The comparison group consisted of individuals that stayed at the same job both years. The likelihood for individuals with no sick leave or 1–180 days of sick leave being employed with regard to job-to-job mobility was also calculated for use as comparisons. Confounders were introduced additively in six models. In Model 1, adjustment was made for age. In Model 2, adjustments were also made for sector and industry. In Model 3, the number of children living at home and marital status were added to the previous confounders, and in Model 4, income and education were added. In Model 5, sick leave the year before the base year was added. Mobility in the year before the base year was added to the other confounders in Model 6. All analyses were conducted separately for men and women and for the various sick leave strata. A 95% confidence interval (CI) was computed for each OR. All analyses were performed using IBM SPSS software (version 20).

## Results

### Descriptives

In all three cohorts, the proportions in employment 3 years after being mobile differed with regard to sickness absence. Men and women show similar patterns. A greater proportion of individuals with more than 180 days sick leave who changed job were employed 4 years later compared with individuals who did not change job (p < .05) (Table 2). Among individuals with 1–180 days sick leave, the proportion of non-employed and employed were similar among those who changed workplace and those who did not. Among individuals with no sick leave who changed workplace, a greater proportion were without employment 4 years later than among those that did not change (p < .05) (Table 2).

**Table 2:** Number and percentage of employed and non-employed in 1998, 2003 and 2008 with regard to job-to-job mobility

**Base year**	**Job mobility**	**Days of sick leave**	**Females**		**Males**	
			**Non-employed 3 years later % ( *n *)**	**Employed 3 years later % ( *n *)**	**Non-employed 3 years later % ( *n *)**	**Employed 3 years later % ( *n *** **cp**
1994	Changed jobs 1994–1995	0	9.6 (15754)	90.4 (148467)*	7.2 (13024)	92.8 (167566)*
		1–180	14.7 (4370)	85.3 (25319)	15.1 (2684)	84.9 (15071)
		181–365	34.9 (583)	65.1 (1086)*	35.5 (440)	64.5 (801)*
	Same job 1994–1995	0	8.2 (76472)	91.8 (860900)	6.2 (64302)	93.8 (980129)
		1–180	14.8 (23403)	85.2 (134892)	14.6 (14540)	85.4 (85362)
		181–365	42.6 (4443)	57.4 (5979)	45.0 (2848)	55.0 (3479)
1999	Changed jobs 1999–2000	0	11.1 (26751)	88.9 (215198)*	9.5 (27118)	90.5 (258911)*
		1–180	14.5 (5741)	85.5 (33921)*	14.8 (3440)	85.2 (19860)
		181–365	33.0 (1091)	67.0 (2217)*	26.1 (361)	73.9 (1022)*
	Same job 1999–2000	0	9.2 (90405)	90.8 (887677)	8.1 (94255)	91.9 (1066225)
		1–180	15.5 (25395)	84.5 (138014)	15.0 (14236)	85.0 (80365)
		181–365	43.8 (7843)	56.2 (10069)	31.9 (1993)	68.1 (4248)
2004	Changed jobs 2004–2005	0	6.4 (12083)	93.6 (176354)*	5.6 (12093)	94.4 (203102)*
		1–180	9.0 (2898)	91.0 (29271)*	9.9 (1673)	90.1 (15296)
		181–365	19.2 (976)	80.8 (4106)*	21.1 (572)	78.9 (2136)*
	Same job 2004–2005	0	5.1 (54906)	94.9 (1016062)	4.2 (51074)	95.8 (1165491)
		1–180	8.9 (14802)	91.1 (151591)	10.0 (8514)	90.0 (76912)
		181–365	23.3 (6449)	76.7 (21200)	25.2 (3283)	74.8 (9740)

*p = <0.05. x^2^, comparing distributions between non-employed and employed among those who changed jobs and those that remained in the same jobs, within each level of sick leave.

In the cohort from 1994, 1998 is the outcome year. In the cohort from 1999, 2003 is the outcome year. In the cohort from 2004, 2008 is the outcome year.

### Multivariate analysis

Women with more than 180 days of sick leave in 1994, 1999 and 2004, and who had changed workplace, were less likely to be unemployed 4 years later compared with those who stayed at the same work place, based on crude data and in the full model. Adjustment for confounders did not affect the ORs. Therefore, only models 1 and 7 are shown (Table 3). Men with more than 180 days sick leave who changed workplace in 1994/1995 or in 2004/2005 were also more likely to be employed 4 years later, based on data adjusted for age and in the full model. However, men with more than 180 sick leave days in 1999 who changed job did not have a higher likelihood of remaining in work than men who did not change job.

**Table 3:** **Odds ratios (OR) and 95**% **confidence intervals (95**% **CI) for unemployment 4 years after a change of workplace among men and women; people who did not change workplaces are the reference category**

**Days of sick leave at the base year**									
	**1994 OR [95% CI] ( *n *)**			**1999 OR [95% CI] ( *n *)**			**2004 OR [95% CI) ( *n *)**		
	**0**	**1–180**	**181–365**	**0**	**1–180**	**181–365**	**0**	**1–180**	**181–365**
Females (model 1)	1.27 [1.25,1.29]	1.13 [1.09,1.17]	0.76 [0.68,0.84]	1.36 [1.34,1.39]	1.10 [1.06,1.14]	0.65 [0.60,0.70]	1.40 [1.37,1.43]	1.18 [1.13,1.23]	0.88 [0.81,0.95]
Females full model (model 7)	1.24 [1.22,1.27]	1.14 [1.10, 1.19]	0.73 [0.65, 0.83]	1.28 [1.25,1.30]	1.08 [1.04,1.12]	0.60 [0. 55, 0.66]	1.28 [1.25,1.31]	1.12 [1.07,1.18]	0.79 [0.72, 0.86]
Males (model 1)	1.35 [1.32,1.38]	1.24 [1.18,1.30]	0.72 [0.63,0.82]	1.52 [1.49,1.54]	1.29 [1.24,1.35]	0.95 [0.83,1.10]	1.60 [1.56,1.63]	1.18 [1.11,1.25]	0.92 [0.83,1.02]
Males full model (model 7)	1.27 [1.24,1.30]	1.16 [1.10,1.23]	0.66 [0.56, 0.77]	1.42 [1.39,1.44]	1.20 [1.14, 1.27]	0.88 [0.75, 1.04]	1.45 [1.42,1.49]	1.10 [1.03,1.17]	0.82 [0.73, 0.92]

Model 1, days of sick leave + age; model 7, model 1 + sector, industry;+ children, marital status + education, disposable income + sick leave year before base year + job change year before base year + rate of sickness absence.

Females and males with no sick leave or 1–180 days of sick leave in 1994, 1999 and 2004 who had changed workplace were, unlike those with the highest level of sick leave, less likely to be employed 4 years later compared with those who stayed in the same job. As in the analysis on individuals with more than 180 days of sick leave, adjustment for confounders did not affect the ORs (not shown) (Table 3).

The analyses (full model) were also done 2 and 3 years after the base years. With 1994 as the base year, both females and males with more than 180 days sick leave who had changed workplace had a decreased likelihood for unemployment in 1996 and 1997 compared with people who stayed at the same work place. With 1999 as the base year, females with more than 180 days sick leave who had changed workplace had a decreased likelihood for unemployment in 2001 and 2002. For males in the cohort from 1999, this was only true in outcome year 2001, in outcome year 2002 the association was not statistically significant. With 2004 as the base year, individuals with more than 180 days of sick leave who had changed workplaces had a decreased likelihood for unemployment in 2006 and 2007, except for males in outcome year 2006, when the association was not statistically significant.

## Discussion

The aim was to investigate whether job-to-job mobility affected the likelihood of men and women with a history of long-term sick leave remaining on the labour market. The results show that females had an increased probability of being in employment 2–4 four years later if they had changed jobs compared with if they had not. Among men with high sickness absence, job change was associated with the future vocational situation in the cohort from 1994 and in the cohort from 2004. Among individuals with a history of 1–180 days sick leave and among individuals without a history of sick leave, changing jobs was associated with a decreased probability of being in employment later.

There is a lack of previous studies on the association between job-to-job mobility and the future vocational situation for individuals with a history of long-term sick leave with which our results can be compared. Ekberg et al. [21] suggested that job mobility could be a possible rehabilitation strategy and Sandmark et al. [38] report in an interview study that mobility in working life could counteract sick leave and contribute to retained work ability.

### Individuals with no sick leave or 1–180 days sick leave

A finding that was not the focus of this study but which is still noteworthy is that individuals with no history of sick leave had an increased probability of unemployment after a job change. One reason might be that job change is associated with fixed-term contracts and part-time work. Sixteen percent of all those employed in Sweden had temporary employment in 2008. Individuals with temporary employment are more likely to have experienced multiple workplace changes. Moreover, those with temporary employment contracts are more likely to move from employment to unemployment [28]. In an attempt to take into account the type of employment contract, earlier mobility between 2003 and 2004 was introduced as a confounder. The correlation between mobility and not having employment for individuals with no history of sick leave weakened, but was still statistically significant. This indicates that some of the individuals who changed jobs and ended up non-employed could belong to a group of frequent job changers.

In Sweden, the purpose of the Employment Protection Act is to protect employees in cases of termination or dismissal. This law includes rules for turn-taking and states that employees with a longer length of service have priority over employees with a shorter length of service, if they are qualified enough. For individuals who have recently changed workplaces, this could be a disadvantage. For this reason, one possible explanation why mobility increases the likelihood of being out of work for individuals without a history of sick leave is that in the case of dismissals, the most recently employed is the first one to be dismissed owing to legislation. If this is correct, we could expect another result if the same study is performed in a place where the law is different. Another explanation could be that frequent job changes are associated with health risk behaviours such as smoking [39]. Health risk behaviours could lead to worse health in the future and may increase the probability of unemployment. To take into account frequent job changes, mobility the year before the base year was introduced as a confounder. For individuals with between 1 and 180 days sick leave, introducing earlier mobility as a confounder reduced the probability of not having a job later for individuals who changed workplaces, but the association between job change and future unemployment was still statistically significant.

In the light of the possible explanations above, the results of the analysis of individuals with no history of sick leave seem plausible. However, these possible explanations do not seem to be valid for individuals with a history of long-term sick leave. Job change among those with many days of sickness absence and those with no such absence may represent different strategies. Among those with no sickness absence, job change may represent an unstable position on the labour market or attempts to seek better circumstances. For those with many days of sickness absence, job change is likely to represent a strategy to obtain a job situation that better matches the individual’s health in order to stay on the labour market.

### Individuals with more than 180 days sick leave

Individuals with long-term sick leave who changed jobs may have better health than those who did not change jobs and therefore had better conditions not to be non-employed, which may explain why job change is associated with an increased likelihood of staying in employment. An attempt to take into account eventual differences in health between those who changed jobs and those who did not was made by adjusting for days of sick leave the year before the base year, assuming that those with worse health had a longer history of sick leave. However, adjustment for earlier days on sick leave did not affect the results.

It is possible that the increased probability of remaining on the labour market among individuals with long-term sick leave was underestimated. As described earlier, those with a temporary employment contract are more likely to change jobs. They are also more likely to move from employment to unemployment [28]. Temporary employments are also associated with poor health [40]. As those with a temporary employment contract are more likely to change jobs it is likely that a higher proportion of those who had changed jobs in this study had temporary jobs. Information about temporary employment contracts was not available. In an attempt to take into account the type of employment contract, earlier mobility was introduced as a confounder, but this had a marginal effect.

Among individuals classified as not mobile, there might be some who were internally mobile, that is, they changed tasks within the workplace. Information on internal mobility is lacking. If such mobility increases the likelihood of staying in work, our results on individuals with a history of long-term sick leave will be underestimated.

Adjustment latitude, that is, opportunities to adjust work to health, increases the likelihood of return to work for people on long-term sick leave [41]. It is possible that the individuals who stayed in the same job had greater adjustment latitude or got more workplace interventions at the current workplace and therefore had no need to change workplaces. If those who did not change jobs had greater adjustment latitude, our results might be underestimated. It is also possible that moving to a new job with greater adjustment latitude is a reason for mobility, which could partly explain our findings. Future studies that include internal mobility and adjustment latitude are required. The probability of men with high levels of sick leave being employed for 1999 was not affected by whether they changed jobs or not. A possible explanation why job mobility affects future employment status differently among men and women may be that men have more opportunities to adjust work to health than women [41]. Thus males in the control group who did not change workplace may be able to stay employed to a greater extent than females in a comparable situation due to better opportunities to adjust work to health.

The effect of job-to-job mobility on the likelihood of people with many days of sick leave staying on the labour market might differ in welfare systems with different rules and different incentives for mobility. However, in this study a similar pattern emerged over a time period of 15 years, despite variations in sick leave and unemployment levels.

### Methodological considerations

The cohorts studied only comprise those having employment in each base year and the following year. To be able to identify job change, the individual has to be employed in both years. Job change is defined as a change of employment number. As there is only one employment number each year, it is not possible to use only one year; job change has to be defined as a change from one year to another. Another reason for setting up this criterion is to enable comparisons with individuals that are employed at the same work place both years, that is, the non-mobile. Owing to a selection of individuals with employment in both years in the mobile group but not in the comparison group, our results may have been overestimated. If this criterion was not set up, job change would have appeared more favourable as all individuals in the job change group had employment in both years, which could indicate, for example, better health, higher motivation or self-efficacy, factors that could predict remaining on the labour market. It is still possible that the results might be due to differences in the group that we were not able to take into account. We have tried to account for differences in health by using earlier days of sick leave as a confounder (see above under *Individuals with more than 180 days sick leave*). In future studies, the role of factors such as motivation and self-efficacy should be considered.

Long-term sick leave is associated with unemployment and disability pension [7-10]. As the study concerns job-to-job mobility and not mobility in and out of the labour market, the majority of the long-term sick are excluded from the cohorts. Males are excluded to a greater extent than women, especially in the cohorts from the 1990s. This might be due to that fact that the employment rate in the Swedish population in the latter half of the 1990s was comparatively low. The employment rate among men decreased more than the employment rate among women [42].

Gainfully employed was classified as working at least 1 hour a week in November. Although a test was conducted in which the proportions of other income sources were compared between those classified as gainfully employed and those not, showing that those classified as not gainfully employed had other income sources such as unemployment benefits during the year to a much higher degree, it is possible that some individuals have been classified as gainfully employed that worked only 1 hour a week in November. This could mean that our results are underestimated. November was chosen with the aim of minimizing the number of individuals engaged in seasonal work, which is more common in summertime.

The analyses were carried out separately for women and men. The difference between men and women is not statistically significant. We still choose to show the results separately as we believe that men and women have different life and working conditions and that different models may therefore affect their chances in working life.

One strength of this study is that the material allows a longitudinal design with identical data for several years. By choosing three cohorts from every fifth year, we were able to consider potential economic and market condition effects. We also wanted to increase the validity by avoiding an outcome caused by chance and certain specific condition in a particular year. In addition, sick leave has varied during these years, which can result in the composition of those listed as sick differing in the groups.

Another strength is the extensive material with information on almost the entire Swedish population. The annual gathering of information on sick leave and workplace is required by law and this is likely to increase reliability and contribute to the small attrition rate. The report on sick leave is also connected to payment of sickness benefits, which increases the reliability of the report. Information on income may have deficient validity as income data were collected for the same year as the sick leave data. Disposable income includes allowances and compensation, but the number of days of sick leave is still likely to affect income, which means that individuals with many days of sick leave were placed somewhat below their normal income level. As a test, income from the year before the base year was used as a confounder instead of income at the base year. This did not alter the results. In future studies, a more valid measurement of income level is desirable.

In the present study, mobility between workplaces was studied. Such mobility may involve moving to a similar job or to a different job and might be perceived as a change to a more or less preferable job. The individual may also have different incentives for changing jobs. Disabled workers are more likely to experience involuntary job changes than nondisabled workers [43]. Such differences in mobility may affect the likelihood of remaining on the labour market. Thus these dimensions should be included in future studies.

### Implications

The results of this study imply that job change might be a way of remaining on the labour market for individuals with a history of long-term sick leave, particularly women. The inclusion criterion in this study, however, limits the validity of the results for individuals with an attachment to the labour market (employment for two years in a row). Furthermore, there might be situations where a job change may not be viable. There might be obstacles that hinder mobility among people who have experienced long-term sick leave. In a Swedish study from 2008, six out of ten personnel managers had a negative attitude about hiring people on sick leave from another workplace [44]. Individuals on long-term sick leave report that their sick leave negatively affects their career and opportunities to change to another job [5]. The results of this study support the hypothesis that job change is a viable strategy for people with a history of long-term sick leave to remain on the labour market. If our results are correct, this would indicate that welfare and labour market policies should be designed to facilitate job change for people on sick leave. “Flexicurity”, which describes a balance between a flexible labour market with a high level of mobility and high levels of social security, has been adopted by the European Union as a strategy for mobility and integration on the labour market. By having flexible and reliable contractual arrangements, employers are supposed to be more willing to employ people with, for example, physical disabilities. By having an adequate and sustainable social protection system, individuals will be protected from job loss [45]. However, as discussed above, the study has methodological limitations and the results for men are ambiguous. We do not therefore have enough evidence to be able to recommend job change as a strategy for vocational rehabilitation.

## Conclusion

Research on the importance of job-to-job mobility among individuals with many days of sick leave is still scarce. The present study indicates that those on long-term sick leave who change workplaces, have better possibilities to stay on the labour market. Since this study is, to the best of our knowledge, the first one to investigate how job change affects the likelihood to remain on the labour market, more evidence is needed before any guidance can be given. In future studies, better measurements of differences in health, type of employment contract and other variables that might affect the conclusions in this study are desirable.

## Competing interests

Funding has been received from AFA Insurance. The authors declare that they have no competing interests.

## Authors’ contributions

KN participated in the design of the study, performed the statistical analyses, contributed to data interpretation and drafted the manuscript. KE contributed to the design of the study and the interpretation of data and participated in drafting the manuscript. TH contributed to the design of the study and the interpretation of data and participated in drafting the manuscript. GJ initiated the study, participated in the design, supervised KN in the statistical analyses and contributed to the interpretation of the data and drafting the manuscript. All authors read and approved the final manuscript.

## Pre-publication history

The pre-publication history for this paper can be accessed here:

http://www.biomedcentral.com/1471-2458/14/305/prepub
